# A Systematic Review on the Impact of Seasonality on Severe Mental Illness Admissions: Does Seasonal Variation Affect Coercion?

**DOI:** 10.3390/healthcare11152155

**Published:** 2023-07-28

**Authors:** Ioannis Rizavas, Rossetos Gournellis, Phoebe Douzenis, Vasiliki Efstathiou, Panagiota Bali, Kostas Lagouvardos, Athanasios Douzenis

**Affiliations:** 1 Psychiatric Hospital of Attica “Dafni”, 12462 Chaidari, Greece; giannisriz@med.uoa.gr; 2 Second Department of Psychiatry, Medical School, University General Hospital “Attikon”, National and Kapodistrian University of Athens, 12462 Chaidari, Greece; rgourn@med.uoa.gr (R.G.); thandouz@med.uoa.gr (A.D.); 3 Medical School, University of Nottingham, Nottingham NG7 2UH, UK; mzypcd@nottingham.ac.uk; 4 Postgraduate Program “Liaison Psychiatry Integrative Care of Physical and Mental Health”, School of Medicine, National and Kapodistrian University of Athens, 12462 Chaidari, Greece; vefstathiou@psych.uoa.gr; 5 National Observatory of Athens, Institute for Environmental Research and Sustainable Development, 15236 Athens, Greece; lagouvar@noa.gr

**Keywords:** seasonality, severe mental illness, psychiatric admissions, involuntary admissions, aggression, coercion

## Abstract

Coercion in psychiatry is associated mainly with involuntary admissions. The purpose of this study was to investigate the associations between hospital admissions of patients suffering from affective and schizophrenic disorders and seasonality. A systematic literature search using PubMed, Scopus and Google Scholar was conducted, including studies with affective and schizophrenia disorder admissions, published from October 1992 to August 2020. A total of 31 studies were included in the review. Four broad severe mental illness admission categories were identified regarding seasonality: affective disorders, schizophrenia disorders, involuntary admission affective disorders and involuntary admission schizophrenia disorders. There was clear and strong evidence for spring and summer peaks for severe mental illness admissions; data provided for age, gender and involuntary admissions was limited. Seasonality may have a significant effect on the onset and exacerbation of psychopathology of severe mental illness and should be considered as a risk factor in psychiatric admissions, violence and the risk of mental health coercion. A better understanding of the impact of seasonality on severe mental illness will help professionals to provide the best practices in mental health services in order to reduce and prevent psychiatric hospitalizations (especially involuntary admissions) resulting in further coercive measures.

## 1. Introduction

In ancient Greece, Hippocrates, in the work “About air, water and places”, analyzes the effect that environment and climate can have on human health. The human body is an integral part of the environment. Therefore, quality of life combined with environmental and climatic factors may affect people’s health. According to this theory, health is defined on the basis of an equilibrium achieved between environmental forces on the one hand (wind, temperature, water, earth and food) and individual habits on the other (diet, alcohol and sexual behavior, and also work and leisure) [1]. Research evidence shows that, in general, winter mortality rates are significantly higher than summer rates [2]. It is well recognized that the season of the year exerts an influence on some diseases and causes of death [3], such as coronary [4,5] and ischemic [6] heart diseases, stroke and cerebrovascular diseases [7,8], respiratory and infectious diseases [9,10], diabetes mellitus [11] and infections in surgical patients [12,13,14].

With regards to the association between seasonality and mental health, the majority of studies have demonstrated significant associations between seasonality and mental disorders [15,16,17,18,19]; a few studies have shown only minimal and limited seasonal differences or have failed to demonstrate a seasonal pattern [20,21,22]. For severe mental illnesses, most studies have shown a significant spring/summer seasonal pattern [23,24]. The impact of seasonality on severe mental illness is well known, such as recently demonstrated in suicidal behaviors [25,26]. In contrast, evidence for the absence of systematic seasonal variation is rather limited [27]. It is likely that seasonality may have an important role in the psychopathology of severe mental illness and may be an influencing factor in hospital admissions [28,29].

Many studies confirm the association between violence and severe mental illness [30,31,32,33]; bipolar and schizophrenia disorders are important causes of involuntary admissions worldwide [34,35]. Involuntary admissions are associated with violent incidents [36,37], and the unavoidable resulting coercion [38] affects both patients [39,40] and mental health personnel [41,42].

However, the methodology in these studies varies, and it is often hard to quantify the findings of the published reports. A systematic review of the impact of seasonality on severe mental health admissions would be useful to policymakers, researchers and healthcare providers to allocate specific resources and provide targeted interventions. In this respect, seasonality should be considered in an attempt to predict the potential dangerousness of patients with affective and schizophrenia disorders [43]. Meteorological predictions might be important in deciding staffing levels and preparations that will result in reducing violent incidents and coercion.

Consequently, the objectives of the review were to: (i) determine whether seasonal conditions are associated with the admission (total, first, readmission) of patients suffering with affective disorders and schizophrenia, (ii) identify the gender and age differences of patients suffering with affective disorders and schizophrenia on admissions in respect to seasonality and (iii) determine the link between involuntary admissions of individuals suffering from affective and schizophrenia disorders and seasonality.

## 2. Materials and Methods

We performed a systematic review of the literature in Scopus and PubMed using various combinations of the key words “seasonality”, “weather”, “meteorological variables”, “severe mental illness”, “affective disorders”, “schizophrenia disorders”, “psychiatric admissions”, “involuntary admissions”. This search was run until 27 January 2021. We developed a data extraction sheet and refined it accordingly. Titles and abstracts of the articles were screened, and the full texts of the selected articles were reviewed and assessed by two researchers independently (I.R. and A.D.) regarding relevance to our inclusion and exclusion criteria. In cases of doubt, consensus meetings (I.R., A.D. and R.G.) reached a decision.

We included 31 studies, written in English, published from October 1992 to August 2020, that investigated the impact of seasonality on severe mental illness hospitalization, for a study period of at least two years and provided information for patients with affective and schizophrenia disorders, aged 15 years old or more, admitted either voluntary or involuntary to psychiatric hospitals or general/university hospital psychiatric units. In these studies, the year was divided into four seasons of equal lengths, namely, spring (March to May—North Hemisphere, September to November—South Hemisphere), summer (June to August—North Hemisphere, December to February—South Hemisphere), autumn (September to November—North Hemisphere, March to May—South Hemisphere) and winter (December to February—North Hemisphere, June to August—South Hemisphere). We excluded systematic reviews that obtained studies that did not comply with the above criteria. Studies that did not examine the impact of seasonality were excluded. The study followed the PRISMA guidelines [44].

The studies varied in setting, population, country of origin and the exposure metrics used. We included articles from 19 different countries (in alphabetical order: Australia, Austria, Brazil, Canada, Czech Republic, Denmark, Egypt, England, India, Israel, Italy, Korea, Norway, Poland, Republic of Ireland, Scotland, Taiwan, United States of America and Wales); of these studies, seventeen were located in the Northern and two in the Southern Hemisphere. Seventeen studies used national or regional mental health register datasets and fourteen used admission data of the respective hospitals included.

## 3. Results

**Figure 1:**
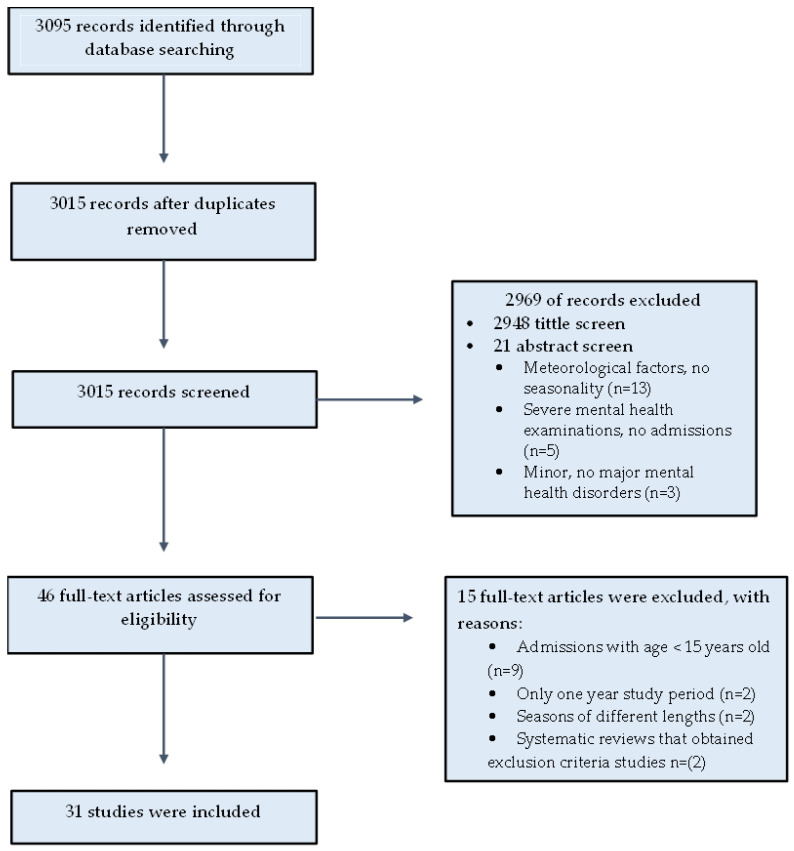
PRISMA flow chart.

A total of 3095 papers were identified; 3015 remained after removal of duplicates. Of these, 2948 were excluded at title and abstract review as they were not within the scope of this review. A total of 67 papers’ full texts were reviewed, of which 31 were included in the study as the remaining did not meet the inclusion criteria (Figure 1).

This systematic review included 31 cross-sectional studies that investigated the association between seasonality and four broad severe mental illness admissions categories: (i) affective disorders total admissions, (ii) schizophrenia disorders total admissions, (iii) affective disorders involuntary admissions and (iv) schizophrenia disorders involuntary admissions (Table 1).

Twenty-five studies described the association between seasonality and admissions with affective disorders [45,46,47,48,49,50,51,52,53,54,55,56,57,58,59,60,61,62,63,64,65,66,67]: (i) seven with bipolar disorder [45,48,49,50,63,66,67], (ii) 22 with manic episodes [29,29,45,46,47,49,50,51,52,53,54,55,56,58,59,60,61,62,63,64,65,66,67], (iii) one with hypomanic episodes [60], (iv) four with mixed episodes [47,51,66,67], (v) eight with bipolar depression [29,50,51,54,57,58,66,67] and (vi) eight with unipolar depression [29,45,48,49,50,51,54,58].

Twelve studies described the association between seasonality and schizophrenia admissions [29,30,45,48,49,50,63,68,69,70,71,72] and three with schizoaffective admissions [50,63,70].

One study described the association between seasonality and involuntary admissions with affective and schizophrenia disorders [28].

Table 2 presents the peaks of admissions for affective and schizophrenia admissions (total, first and readmissions).

### 3.1. Affective Disorders

#### 3.1.1. Bipolar Disorder

Seven studies described the relationship between seasonality and bipolar disorder admissions [45,48,49,50,63,66,67]. In particular, of the four studies investigating total admissions [45,50,66,67], Whitney et al. [66] found significantly more admissions in summer (*p* < 0.05), Yang et al. [67] noticed significantly more admissions in spring (*p* < 0.001), Daniels et al. [50] mentioned a not significant peak in late spring and early summer, while Aguglia et al. [45] reported a slightly higher admission prevalence in spring.

Furthermore, two studies investigating first admissions [48,63], found significant seasonal peak of admissions in summer (August, *p* < 0.001), with a further peak in June [63], whereas Clark et al. [49] exploring readmissions, showed significantly more readmissions in summer (July, *p* < 0.001).

Yang et al. [67] examined the seasonality of bipolar disorder associated with sex and age. Both sexes appeared to have a significant spring (May, *p* < 0.001) peak. Age group analysis showed a significant summer peak (June, *p* < 0.001) for young adults (18–34) and a significant spring peak (April, *p* < 0.001) for middle-aged adults (35–55).

#### 3.1.2. Hypomanic Episodes

Parker and Graham [60] described the relationship between seasonality and hypomanic episodes total admissions. These admissions were at their highest in spring and at their lowest in autumn, while the monthly percentage admission scores indicated lower rates in the first six months of the year (January–June), with a sudden increase in July, followed by a more gradual increase until December.

#### 3.1.3. Manic Episodes

Twenty-two studies described the relationship between seasonality and manic episode admissions [29,45,46,47,49,50,51,52,53,54,55,56,58,59,60,61,62,63,64,65,66,67]. Of these, eighteen studies investigated total admissions [29,45,46,50,51,52,53,54,56,58,59,60,61,62,64,65,66,67]. Bakstein et al. [29] and Dominiak et al. [51] found significantly increased rates in summer (August, *p* < 0.001, *p* < 0.03), with further significantly increased rates in winter (January) and spring (May) (both *p* < 0.03) [51]. Yang et al. [67] mentioned a significant spring peak (*p* < 0.001), Aguglia et al. [45,46] reported a significantly higher prevalence during spring/summer; however, when a stepwise logistic regression analysis was applied, manic episodes no longer predicted this pattern [46]. Jones et al. [53] and Medici et al. [56] found spring/summer [53] and summer (August) [56] peaks. Three studies from Australia [59,60,61] suggested that admissions were higher in spring and lower in autumn and winter. On the contrary, one study from India [62] and two studies from Brazil [64,65] mentioned a significant peak in late winter, minimum in late summer and highly significant evidence for a 3-month peak of admissions between November and January (*p* < 0.01), whereas Whitney et al. [66] indicated a higher prevalence of admissions in autumn. Finally, results from four studies [50,52,54,58], reported no significant seasonal variation.

It is noteworthy that in five studies investigating first admissions [47,49,53,55,63], Clarke et al. [49] and Takei et al. [63] found a significant seasonal peak in summer (August, both *p* < 0.001), with a further peak in June [63], whereas Cassidy et al. [47] and Lee et al. [55] found a significant peak in spring (March) [47,55] and autumn (October) [55]; the lowest incidence was in autumn (November) and winter (January) [55]. One study [53] found no seasonal pattern, whereas investigating readmissions reported a spring peak.

Eight studies [50,51,53,54,58,62,66,67] examined the difference between female and male admissions (total, first admissions and readmissions). As regards females, Dominiak et al. [51] found a significant peak in winter (January, *p* < 0.03), whereas Kerr-Correa et al. [54] reported a significant spring/summer peak (*p* < 0.05). Whitney et al. [66] noticed a peak in autumn and Jones et al. [53] mentioned a peak in spring. Three studies found no significant difference in females [50,58,62]. Dominiak et al. [51] and Morken et al. [58] found significant summer (August, *p* < 0.05) [44] and spring [58] peaks (*p* < 0.01) among males. Rajkumar and Sarkar [62] reported a significant peak for males in winter (December and January, *p* < 0.005), whereas Whitney et al. [66] noticed a preponderance of admissions in autumn. Two studies reported no significant difference in males [50,54]. Yang et al. [67] found a significant peak (*p* < 0.001) for both sexes in spring, with the lowest number of admissions for both sexes observed in autumn (November, *p* < 0.03) [51].

Three studies [53,62,67] also examined the difference between age groups and seasonality. Rajkumar and Sarkar (2015) [62] found a strong evidence for seasonal variation in patients aged over 25 years, with a significant 2-month peak in winter (December and January, *p* < 0.005) and a 3-month peak from late autumn to middle winter (from November to January, *p* < 0.01), there was no significant evidence in patients below the age of 25. Yang et al. [67] found a significant summer peak (June, *p* < 0.001) for young the adult (18–34) group and a significant spring peak (March, *p* < 0.001) for middle adults (35–55), whereas Jones et al. [53] mentioned a spring peak in patients over 50 years old and no seasonal pattern for individuals aged under 30 and in the 30–49 age group.

#### 3.1.4. Mixed Episodes

Of the three studies describing the relationship between seasonality and mixed episodes total admissions [51,66,67], Whitney et al. [66] noted admissions being statistically significantly higher in the summer (*p* < 0.01), Yang et al. [67] found significant peaks for admissions in spring and summer (*p* < 0.001), whereas Dominiak et al. [51] reported most admissions were in late spring (from May to June) and winter, with significantly lower admission rates in autumn (November, *p* < 0.03).

Furthermore, Cassidy et al. [47], investigating first time psychiatric admissions, showed a zenith in late summer (August) and a nadir in late autumn (November).

Three studies [51,66,67] also examined the difference between female and male admissions. In particular, Yang et al. reported a significant spring peak (May, *p* < 0.001) for females and a significant summer (June, *p* < 0.001) peak for males [67]. Dominiak et al. [51] found three periods with higher admission rates for male patients, a peak in February, from May to June, and a small peak in August. The lowest rates of admissions were noted in spring (April, *p* < 0.01) and summer (July, *p* < 0.01). Whitney et al. [66] mentioned that females had a statistically significant peak of admissions in the summer months (*p* < 0.01).

#### 3.1.5. Bipolar Depression

Eight studies described the relationship between seasonality and bipolar depression total admissions [29,50,51,54,57,58,66,67]. Bakstein et al. [29], Dominiak et al. [51] and Morken et al. [58] found admissions peaked significantly in spring (April, *p* < 0.001, *p* < 0.05 and March, *p* < 0.05), with the lowest point in summer (August, *p* < 0.05) [51]. Whitney et al. [66] described no significant preponderance of admissions in spring and summer. Yang et al. [67] reported a significant autumn peak, while Daniels et al. [50] showed an almost significant trend (*p* = 0.05), with a low number of admissions in the autumn. Contrary to this, Modai et al. [57] noticed that during winter the admission rate increased, whereas Kerr-Correa et al. [54] found no significant seasonal pattern.

Furthermore, four studies [50,51,58,67] examined the differences between male and female admissions. Yang et al. [67] reported a significant autumn peak for females (November, *p* < 0.001) and no significant autumn peak for males. Morken et al. [58] mentioned a significant spring peak for females (April, *p* < 0.05) with a nadir in autumn (November) and no seasonal pattern for males. Dominiak et al. [51] reported that the highest rates of admission for males aged between 36 and 65 years were in winter (February, *p* < 0.01) and autumn (November, *p* < 0.05), while the lowest rates were during late spring/summer (from May to August, for all months *p* < 0.01). On the contrary, Daniels et al. [50] showed no statistical significance in the rates of seasonality in admissions related to gender.

#### 3.1.6. Unipolar Depression

Eight studies described the relationship between seasonality and unipolar depression admissions [29,45,48,49,50,51,54,58]. In particular, of six studies investigating total admissions [29,45,50,51,54,58], Bakstein et al. [29], Dominiak et al. [51] and Morken et al. [58] found significant admission peaks in spring, one peak in April (*p* < 0.001, *p* < 0.01) [29,58], another peak in March (for recurrent depression, *p* < 0.05) and May (for a single depressive episode, *p* < 0.03) [51] and a further significant peak in autumn (November, *p* < 0.001) [29,51], with significantly lower rates in summer (August, *p* < 0.001) [29] and winter (December for recurrent depression, *p* < 0.001) [51], while Aguglia et al. [45] noticed a slightly higher rate of admissions in autumn/winter. On the other hand, Daniels et al. [50] and Kerr-Correa et al. [54] reported no significant seasonal variation.

Regarding studies investigating first-time psychiatric admissions, both studies [48,49] showed a significant seasonal peak in summer (August, *p* < 0.001).

Moreover, Morken et al. [58] found a significant spring (April, *p* < 0.05) peak for males, a significant autumn (November, *p* < 0.05) peak and a non-significant spring (April) peak for females, while Daniels et al. [50] showed no significant seasonal variation by gender.

### 3.2. Schizophrenia Spectrum Disorders

#### 3.2.1. Schizophrenia

Twelve studies described the relationship between seasonality and schizophrenia disorder admissions [29,30,45,48,49,50,63,68,69,70,71,72]. Of the eight studies exploring total admissions [29,30,45,49,50,69,70,72], significant summer peaks were reported in Bakstein et al. [29] (*p* < 0.001), Hinterbuchinger et al. [69] (*p* < 0.0001) and Shiloh et al. [70] (*p* < 0.001), with significant monthly peaks in June [29,69], and a further significant peak in winter (January, *p* < 0.0001) [69]. Tian et al. [72] found seasonality in admissions peaking annually in spring (March), Aguglia et al. [45] reported slightly higher rate of admissions in autumn/winter, while Amr and Volpe [30] Clarke et al. [49] and Daniels et al. [50] found no significant seasonal variation.

Furthermore, five studies investigated first admissions of schizophrenia disorder [48,49,63,68,71]. Clarke et al. [48,49] and Takei et al. [63] found a significant seasonal variation in summer (July, *p* < 0.001), Takei and Murray [71] showed a significant peak in spring (May, *p* < 0.005), whereas Davies et al. [68] suggested that individuals were more likely to have their initial admission during winter. Notably, Clarke et al. [49] investigated readmissions, but did not find evidence of seasonal variation.

Moreover, four studies examined the association between gender and total and first admissions [63,68,69,71]. Hinterbuchinger et al. [69] found that total admissions for males and females showed a significant winter (January) and summer (June) peak (both *p* < 0.0001); the largest though was found in December for both sexes. Importantly, out of the three studies exploring first admissions [63,68,71], Takei et al. [63] and Takei and Murray [71] found a highly significant summer (July, *p* < 0.001) [63] or spring (May, *p* < 0.005) [71] peak for females, but not for males, whereas Davies et al. [68] found for both sexes a summer peak (August), which was more pronounced for males.

#### 3.2.2. Schizoaffective Disorder

Three studies investigated the relationship between seasonality and schizoaffective disorder admissions [50,63,70]. Of the two studies exploring total admissions [50,70], Shiloh et al. [70] reported that admission rates were significantly higher during autumn (October and November, *p* < 0.001), while Daniels et al. [50] found a not significant peak in the winter. Takei et al. [63], exploring first admissions, reported no significant seasonal variation.

Two studies examined the association between gender and total and first admissions [50,63]. Daniels et al. [50] mentioned a significant (*p* < 0.01) winter peak for males and a similar but not significant peak for females, while Takei et al. [63] showed no significant seasonal variation for both sexes.

### 3.3. Involuntary Admissions

Aguglia et al. [28] reported that involuntary hospitalizations compared to voluntary admissions occurred particularly in spring (28.6% involuntary versus 21.7% voluntary) and summer (29.5% involuntary versus 21.5% voluntary) (*p* = 0.038). The diagnoses were (i) bipolar and related disorders (50.0% involuntary versus 31.4% voluntary) and (ii) schizophrenia spectrum disorders (36.6% involuntary versus 24.4% voluntary) (*p* < 0.001).

## 4. Discussion

This review summarizes the evidence for an association between seasonality and severe affective and schizophrenia disorder admissions, describing the size and direction of association where available. To the best of our knowledge, this is the first attempt to provide a systematic literature review on this subject. The heterogeneity of studies (data, study design and statistic methods) restricted us from conducting meta-analyses. The findings from 31 articles (comprising over 1.4 million serious mental illness admissions), published between October 1992 and August 2020, showed evidence of a positive association between spring and summer season and an increased risk for severe mental illness admissions in psychiatric hospitals and psychiatric units.

It must be mentioned that some studies in this review included the same clinical sample: three studies from Italy [28,45,46], one for involuntary admissions [28], one for schizophrenia and affective disorders [45] and one for affective disorders [46]; two studies from Ireland [48,49], both for schizophrenia and affective disorder admissions; three studies from Australia [59,60,61] for mania and hypomania admissions. This does not appear to affect the final conclusions of the review.

The majority of the studies that investigated bipolar disorder admissions found significantly more admissions in summer, one study showed a significant peak in the spring and the remaining studies reported a higher prevalence of total admissions in spring and summer.

Out of twenty-two studies for manic episodes, eight studies found significantly seasonal increased rates for admissions, three in the summer, three in the winter, one in the spring and one with spring/summer/winter peaks. Five studies showed increased peak rates in spring, one in summer, three in spring/summer, one in the autumn and one in spring/autumn. The results from four studies reported no significant seasonal variation. As regards the association between gender and admissions, eight studies examined the impact of seasonality on female admissions. Two reported a significant spring and spring/summer peak, one showed increased rates in the spring, two studies found a different seasonal pattern, while three studies showed no seasonal variation. Two studies mentioned significant peak for male admissions in the spring, one in the summer and one in the winter, whereas two studies reported no significant difference.

Four studies examined the impact of seasonality on mixed episodes admissions; two studies reported significant peaks in the summer and spring/summer and two studies found seasonal peaks in the spring and summer.

Eight studies described the relationship between seasonality and bipolar depression total admissions; three reported that admissions significantly peaked in the spring and the results from the rest of the studies showed a different or no seasonal variation.

Eight studies investigated a possible association between the seasons of the year and unipolar depression admissions; two found a significant peak in summer, two with significant peaks in the spring and autumn, one study found a spring peak, whereas two studies reported no significant difference.

Out of twelve studies regarding schizophrenia admissions, four studies found a significant summer peak, one study found admissions peaking annually in the spring, one study mentioned a significant peak in the spring, another a significant summer/winter peak, while three studies found no significant seasonal variation.

The results of three articles regarding schizoaffective disorder mentioned different seasonal variations; one showed a significant peak in the autumn, one found an admissions peak in winter and one reported no seasonal variation.

There was only one article on involuntary admissions; the majority of patients with bipolar disorder and schizophrenia who were admitted involuntarily were more likely to be hospitalized in spring and summer.

There were limited and varied data regarding the impact of seasonality on admitted patients, by gender and age, so it was not possible to draw firm conclusions.

The mechanisms and possible changes underlying the effects of seasonality on severe mental illness remain unclear. Mental health disorders are multifactorial and possibly influenced by complex interactions between genetics, nutrition and many environmental and social factors, which might increase the risk for severe mental health illness [73]. The nature and intensity of environmental factors and the temporal relationship between symptoms and these environmental factors need further investigation [45]. A positive association and seasonal variation between exposure to ozone and fine particles and all mental health-related emergency department visits is reported, though the underlying biological mechanisms remain poorly understood [74]. Studies have linked schizophrenia and prenatal exposure to a number of microbial infections that may be more common during colder months when the population is more likely to get sick [75].

Three factors may play an important role in bipolar disorder with a seasonal pattern: the suprachiasmatic nucleus, the melatoninergic system and the photoperiodism system. This disorder is considered complex, resulting from an interaction of clock gene vulnerabilities and biological clock neuroplasticity with environmental factors. Circadian rhythms are the daily light–dark cycle that governs physical, mental and behavioral changes. These natural processes respond primarily to light and dark [76]. The clock genes have been reported to regulate circadian rhythms [77]. There is growing evidence that factors such as gene polymorphisms of the core clock machinery and seasonal changes of the light–dark cycle, affecting the biological clock, influence the behavior of patients affected by mood disorders [78]. Other studies have reported an association between genetic variants in circadian clock genes and severe mental illness [79], such as major depressive disorder, bipolar disorder and schizophrenia [80,81]. A change in temperature can affect the phase of a cycle without affecting the rate of cycling on circadian rhythms, which means that the cycle may start earlier or later than normal but will have the same duration [76]. In addition, the Papez circuit, which has been associated with emotional states, has been shown in a study to have melatonin receptors, a finding that might offer a further pathway for researchers to explore [82].

The response to light appears to play a key role due to neural connectivity: a pathway linking light to the cortical serotonin transporter and a pathway linking light to melatonin synthesis [83]. Furthermore, patients with bipolar disorder have a higher light sensitivity, as a result of melatoninergic, serotoninergic and dopaminergic neurotransmission [84]. It is hypothesized that the underlying mechanism for seasonal mania may be hypersensitivity to bright light that suppresses melatonin production [85]; vitamin D deficiency and acute manic episode may have interactions with many pathways [86].

Heat stress can affect psychophysiological and cognitive functions by altering the levels of serotonin and dopamine (heat increases plasma serotonin, which inhibits dopamine production), which are responsible for both thermoregulation and behavioral and mental states, by influencing the function of central and peripheral thermoregulation in the body [87,88,89].

The turnover of serotonin 5HT is lower in autumn and winter [90,91] and higher around the summer solstice [91]. The rate of production is directly related to the prevailing duration of bright sunlight, rapidly rising with increasing luminosity. Changes in the release of serotonin might underlie mood seasonality and seasonal affective disorder [91]. Levels of serotonin 5HT may be associated with variations in sunlight, and it is possible that photoperiod and sunlight variations alter neurotransmitter concentrations that might play a role in triggering manic episodes [59].

The neuroendocrine signaling cascade, called the hypothalamic–pituitary–adrenal (HPA) axis [92], is the primary endocrine response to stress [93]. Researchers have recognized the strong association of dysfunction of HPA axis activity with psychiatric disorders [92,93,94], such as major depression [95]. When cortisol levels increase, corticotropin levels usually decrease, whereas when cortisol levels decrease, corticotropin levels usually increase. The overexpression of corticotropin contributes to HPA axis hyperactivity in psychiatric patients [93]. Cortisol levels show significant seasonal variation, with reduced levels occurring during the summer (long photoperiod) and increased levels during the winter (short photoperiod). Seasonal changes in cortisol secretion could therefore have the effect of a season-dependent modification of the adjustment of biological functions to the environment [96].

Other seasonal social factors, such as recreational use of stimulants, stressful life events, late nights and holidays, may also contribute to the seasonality of mental disorders [97,98]. Modern human activity in air-conditioned spaces (overheated in winter and cooled in summer), reduce the human body’s ability to naturally adapt and respond to different environmental conditions, as the capacity of self-regulating mechanisms are no longer able to respond to weather changes [99].

The aggressiveness of hospitalized patients with severe mental illness correlates with seasonality, as rates of aggression and aggressive behavior peaked in certain months or seasons of the year [100,101,102]. Most of the seclusion incidents and the number of restrained patients showed a circannual rhythm and monthly rates [43,103,104]. The majority of coercive measures concerned males under 50 years old, suffering from substance-abuse, schizophrenia or affective disorders. There was a significant increase in pharmacological coercion during spring and mechanical coercion during summer [105]. Wynn reported a seasonal peak of restraint in autumn [106]. A study from Finland noticed that variation in the prevalence of seclusion and restraint was not consistent with the variance in violence, which implies that the use of coercive measures is related to seasonal variation among staff [107].

Extreme precipitation and temperature have a significant effect on mental health admission [108,109]; heat can cause psychological distress in patients with mental health disorders and lead to alcohol and substance abuse and aggressive behavior [110]. Among involuntary admissions, physical restraint was more prevalent during spring compared to the other seasons [28]. Exploring meteorological factors associated with involuntary hospitalizations could lead to early intervention and prevention strategies for traumatizing hospitalizations [111].

Alterations in serotonin function affected by environmental temperature may cause aggression and suicidal behavior [112,113]. Adrenaline generation is helpful in keeping the human body within safe limits in response to excessive heat, but as a side effect it leads to aggression [114].

As the increasing frequency of extreme events (floods, droughts and heat waves) caused by global climate change are more likely [115], this review underlines that bearing in mind the climate changes, greater attention should be given to seasonal and meteorological parameters that are associated with violent behavior. Meteorological predictions need to be introduced and taken into account as an influential parameter in future mental health policies in order to reduce violent incidents, involuntary admissions and coercive measures.

### Limitations and Strengths

Among the limitations of our study is the exclusion of non-English articles and thus the possibility of relevant studies from other countries not taken into consideration. Furthermore, because more than half of the studies included in this review used older diagnostic criteria (ICD-7 to ICD-9, DSM-III and DSM-IV), it is unclear whether all patients meet the current diagnostic criteria by the ICD-10 or DSM-5.

Additionally, all data obtained were for patients requiring hospitalization, and their seasonal pattern may differ from those not admitted to hospitals. The findings were based on the hospital admission date, rather than the actual onset of the acute episode. Factors that could contribute to the onset of an acute psychiatric episode had not been taken into consideration and could not be ruled out as contributing factors. Most of the studies used national or regional mental health register datasets. Psychiatric diagnoses based on administrative data are considered to be less accurate than those based on face-to-face structured interviews. In addition, index diagnoses may change over time in some patients. In terms of studies considering age and sex, the number of studies was small, making it difficult to draw reliable conclusions. There was only one study for involuntary admissions, thus it was not possible to draw conclusions for this group of patients. Furthermore, the heterogeneity of methods used in studies undertaken to date limits this review to a narrative synthesis without meta-analysis. Lastly, despite the likelihood that meteorological parameters may affect mental health, most studies did not investigate the impact of weather and geographical data.

On the other hand, we included studies published after 1990, intending to present contemporary results and to approach the reality of climate change that humanity has been experiencing over the last decades. Furthermore, the age of admissions was limited to older than fifteen years, having in mind that child and adolescent mental health has different characteristics as well as provided services from those of adults. Of note, another strength of our study is that it followed PRISMA criteria and guidelines.

## 5. Conclusions

This systematic review is the first, to our knowledge, to summarize the impact of seasonality on severe mental illness admissions. The findings showed evidence of a positive spring/summer association. The results have highlighted gaps in the knowledge base and have identified areas relevant to researchers, providers and commissioners, emergency psychiatric departments, outpatient facilities and community services actions.

Although the results of this review suggest a positive relationship between spring/summer and severe mental illness admissions, significant gaps exist for the impact of seasonality, thus requiring further research and data collection. Specific areas that need further research include the impact of seasonality on common mental health illness admissions (neurotic, stress-related and somatoform disorders); further sociodemographic characteristics (age, gender, etc.), for all psychiatric admissions; underlying influencing factors and their mechanisms, such as meteorological parameters (heat, precipitation, atmospheric pressure, humidity, wind, etc.), individually or in combination; the type of hospitalizations (voluntary or involuntary) and aggression and coercive measures.

## Figures and Tables

**Table 1:** Description of studies included in the systematic review.

Authors/Year of Publication	Study Type/Source, Country	Period of Study/Years of Survey	Sample Size/Age	Diagnostic Criteria	Admissions Characteristics	Main Results
[28] Aguglia et al. (2016)	Cross-sectional (retrospective)/Turin, Italy	2013–2015 (2)	112/ mean age (±SD) 43.2 (±13.7)	DSM 5	Sample of affective disorders and schizophrenia involuntary admissions.	Involuntary affective and schizophrenia disorders, significant peak in spring and summer.
[45] Aguglia et al. (2017)	Cross-sectional (retrospective)/Turin, Italy	2013–2015 (2)	730/ mean age (±SD) 43.4 (±13.9)	DSM 5	Sample of affective and schizophrenia admissions.	Bipolar disorder, slightly higher prevalence in spring, manic episodes in spring/summer, unipolar depression and schizophrenia in autumn/winter.
[46] Aguglia et al. (2018)	Cross-sectional (retrospective)/Turin, Italy	2013–2015 (2)	730/ mean age (±SD) 43.4 (±13.9)	DSM 5	Sample of affective admissions.	Manic episodes, significantly increased in spring and summer. Stepwise logistic regression analysis showed manic episode no longer predicted spring/summer pattern.
[30] Amr and Volpe (2012)	Cross-sectional (retrospective)/Mansoura, Egypt	2003–2007 (5)	3346/ mean age (±SD) 27.3 (±5.2)	DSM IV	All affective and schizophrenia admissions.	Manic and mixed episodes, significantly increased in summer (June), unipolar and bipolar depression in winter (December). Schizophrenia, no seasonal variation.
[29] Bakstein et al. (2020)	Cross-sectional (retrospective)/Czech Republic	1994–2013 (20)	231,573/ Not specified	ICD-10	All affective and schizophrenia admissions.	Mania, significantly increased in summer (August), bipolar and unipolar depression in spring (April), schizophrenia in summer (June). Unipolar depression, significantly lower rates in summer (August).
[47] Cassidy et al. (2002)	Cross-sectional (retrospective)/ North Carolina, United States	1993–1996 (3)	304/ 18–82	DSM-III-R	First mixed or manic bipolar admissions.	First manic episodes, peak in early spring (March) and nadir in late fall (November). First mixed episodes, peak in late summer (August) and nadir in late fall (November).
[48] Clarke et al. (1998)	Cross-sectional (retrospective)/Ireland	1989–1994 (6)	13,240/ Not specified	ICD-9 & ICD-10	First schizophrenia and affective admissions.	First bipolar and unipolar depression, significant peak in summer (August), schizophrenia also in summer (July).
[49] Clarke et al. (1999)	Cross-sectional (retrospective)/Ireland	1989–1994 (6)	65,738/ Not specified	ICD-9 & ICD-10	All schizophrenia and affective disorders admissions.	First manic episodes and depressive disorder, significant peak in summer (August), schizophrenia also in summer (July). Bipolar disorder readmissions, significant peak in summer (July).
[50] Daniels et al. (2000)	Cross-sectional (retrospective)/Tasmania, Australia	1983–1989 (7)	8464/ Not specified	ICD-9	All schizophrenia and affective disorders admissions.	Schizoaffective disorder, significant winter peak for males, a similar but not significant peak for total sample and females. Bipolar disorder, not significant peak in late spring and early summer. Bipolar depression, significant low trend in autumn, no statistical significance by gender. Mania, depression and schizophrenia, no significant seasonal variation, either the entire sample or by gender.
[51] Dominiak et al. (2015)	Cross-sectional (retrospective)/Warsaw, Poland	2002–2010 (9)	2837/ >18	ICD-10	Sample of affective disorders admissions.	Mania, significantly increased in winter (January), spring and summer (May and August). Significantly lowest number in autumn (November). For females, significant variation in winter (January), for males in summer (August). Mixed episodes, extra admissions in winter (December to February) and spring/summer (May to June), significant lowest rate in autumn (November). For males, peaks in winter (February), spring/summer and a small extra peak in summer (August), significantly lowest rates in spring (April) and summer (July). Bipolar depression, significant highest in spring (April), significant lowest in summer (August). In males aged 36–65 years, significant highest in winter (February) and autumn (November), significant lowest in spring/summer (from May to August) and in winter (December). Recurrent depression peaks in spring (March) and autumn (November), significant lowest rates in winter (December). Single depressive episode, significant highest number in spring (May), lowest admissions rate late autumn (November).
[52] Jain et al. (1992)	Cross-sectional (retrospective)/Bangalore, India	1980–1988 (9)	270/ Not specified	ICD-9	All mania admissions.	Mania, no seasonal variation.
[53] Jones et al. (1995)	Cross-sectional (retrospective)/Tasmania, Australia	1983–1989 (7)	1280/ Not specified	ICD-9	All mania admissions.	Mania, spring/summer total admissions peaks. Mania, spring readmissions peak, no seasonal pattern for first admissions. Mania, spring peak in female readmissions. Mania, spring peak in patients over 50 years old, no seasonal pattern for under 30 and 30–49 age groups.
[54] Kerr-Correa et al. (1998)	Cross-sectional (retrospective)/Botucatu, Brazil	1982–1991 (10)	157/ Not specified	DSM-III-R & ICD-9	All bipolar disorder admissions.	Mania, no significant seasonal variation for total admissions. Mania, significant spring/summer peak in women, no significant difference in men. Bipolar and unipolar depression, no significant seasonal pattern.
[55] Lee et al. (2002)	Cross-sectional (retrospective)/Seoul, Korea	1996–1999 (4)	152/ 18–33	DSM-III-R	First manic admissions.	Mania, peaks in spring (March) and autumn (October), lowest in winter (January).
[56] Medici et al. (2016)	Cross-sectional (retrospective)/Denmark	1995–2012 (18)	24,313/ ≥15	ICD-8 & ICD-10	All mania admissions.	Mania, summer (August) peak.
[57] Modai et al. (1994)	Cross-sectional (retrospective)/Petah Tikva, Israel	1988–1990 (3)	63/ mean age (±SD) 59.1 (±15.18)	DSM III	All bipolar depression admissions.	Bipolar depression, winter increase.
[58] Morken et al. (2002)	Cross-sectional (retrospective)/Norway	1992–1996 (5)	4341/ ≥18	ICD-9 & ICD-10	All affective disorder admissions	Mania, significant spring peak among men, but not among total sample or women. Bipolar depression, significant spring peak for total sample (March–April) and females (April) with a nadir in autumn (November), no seasonal pattern for males. Unipolar depression, significant spring (April) peak for total sample and males, a significant autumn (November) peak and a non-significant spring (April) peak for females.
[59] Parker et al. (2018)	Cross-sectional (retrospective)/New South Wales, Australia	2000–2014 (15)	21,882/ Not specified	ICD-10	All mania admissions.	Mania, peak in spring, abrupt change in winter (June to August in NSW).
[60] Parker & Graham (2016)	Cross-sectional (retrospective)/New South Wales, Australia	1999–2014 (14)	27,255/ Not specified	ICD-10	All mania and hypomania admissions.	Mania and hypomania, similar pattern, lowest in autumn, increasing in winter, highest in spring.
[61] Parker et al. (2017)	Cross-sectional (retrospective), New South Wales, Australia	2000–2014 (14)	21,882/ Not specified	ICD-10	All mania admissions.	Mania, peak in spring (October and November NSW).
[62] Rajkumar & Sarkar (2015)	Cross-sectional (retrospective)/Pondicherry, India	2010–2013 (4)	357/ Adults	ICD-10	All mania admissions.	Highly significant 3-month peak (November to January, most significant 5-month peak (November to March). Males, significant winter peak (December to January), no peak in females. Patients over the age of 25, significant 2-month peak in December and January, a 3-month peak from November to January. Patients below the age of 25, some evidence for a 5-month peak (November to March), and a 6-month peak (November and April).
[63] Takei et al. (1992)	Cross-sectional (retrospective)/England and Wales	1976–1986 (11)	38,615/ Not specified	ICD-8 & ICD-9	First schizophrenia and affective disorders admissions.	Mania, significant summer peak for total sample (August), males (August) and females (July). Schizophrenia, total and females significant peak in summer (July). Four of the six schizophrenia subtypes, significant summer cyclical trend in females, none in males. Schizoaffective disorder, total sample and both sexes, no significant seasonal variation.
[64] Volpe et al. (2010)	Cross-sectional (retrospective)/Belo Horizonte, Brazil	2000–2007 (8)	5172/mean age (±SD) 41.3 (±12.5)	ICD-10	All manic admissions	Mania, significant peak in late winter, minimum in late summer.
[65] Volpe & Del Porto (2006)	Cross-sectional (retrospective)/Belo Horizonte, Brazil	1996–2000 (5)	425/ mean age (±SD) 42 (±13)	ICD-10	All manic admissions.	Mania, significant peak in late winter, minimum in late summer.
[66] Whitney et al. (1999)	Cross-sectional (retrospective)/Ontario, Canada	1920–1995 (75)	5317/ Not specified	Case conference (1920–1960) ICD-9 (1970–1990)	All bipolar disorder admissions.	Bipolar disorder, significant peak in summer. Mania, total and both sexes preponderance in autumn. Bipolar depression, preponderance in spring and summer, both sexes in autumn. Mixed total and females episodes statistically higher in summer (June).
[67] Yang et al. (2013)	Cross-sectional (retrospective)/Taiwan	2002–2007 (6)	9619/ 18–55	ICD-9	Sample of bipolar disorder admissions.	Bipolar disorder, total and both sexes, significant spring (May) peak. Manic episodes, total and both sexes, significant spring peak. Bipolar depression, significant autumn (November) peak for females, not significant peak for males (September) Mixed episodes, significant spring peak (May) for females and significant summer (June) peak for males. Young adult (18–35), significant summer (June) peak for bipolar disorder, manic and mixed episodes, significant autumn (October) peak for bipolar depression. Middle adult (35–55), significant spring peak for bipolar disorder (April), manic (March) and mixed (May) episodes, not significant winter (December) peak for bipolar depression.
[68] Davies et al. (2000)	Cross-sectional register-based/Queensland, Australia	1973–1991 (19)	7739/ Not specified	ICD-8 & ICD-9	First schizophrenia admissions.	Most admission during winter, both sexes peak in summer (August), seasonality more pronounced for males.
[69] Hinterbuchinger et al. (2020)	Cross-sectional register-based/Austria	2003–2016 (14)	110,735/ ≥15	ICD-10	Sample of schizophrenia admissions.	Significant variation peaks in midwinter (January) and summer (June) and a trough in early winter (December). Hospitalizations follow a seasonal pattern in both men and women
[70] Shiloh et al. (2005)	Cross-sectional register-based/Tel-Aviv, Israel	1981–1991 (11)	33,614/ >18	ICD-9	All schizophrenia, schizoaffective disorder admissions.	Schizophrenia significantly higher in summer. Schizoaffective significantly higher in autumn (October and November).
[71] Takei & Murray (1993)	Cross-sectional register-based/Scotland	1961–1990 (30)	14,964/ Not specified	ICD-7 & ICD-8 & ICD-9	First schizophrenia admissions.	Schizophrenia total and females significant peak in spring (early May).
[72] Tian et al. (2006)	Cross-sectional register-based/Taiwan	1997–2003 (7)	759,611/ ≥18	ICD-9	All schizophrenia admissions.	Schizophrenia peak in spring (March).

**Table 2:** Number of studies testing Significant Associations (SA) or Positive Correlations (PC) of severe mental illness admissions peaks and seasonality.

	Spring		Summer		Autumn		Winter		Spring/Summer		Spring/Autumn		Spring/Summer/Winter		Summer/Winter		Autumn/Winter	
	SA	PC	SA	PC	SA	PC	SA	PC	SA	PC	SA	PC	SA	PC	SA	PC	SA	PC
Bipolar disorder	1 [67]	1 [45]	4 [48,49,63,66]							1 [50]								
Manic episodes	1 [67]	5 [47,53,59,60,61]	3 [29,49,63]	1 [56]		1 [66]		3 [62,64,65]		3 [45,46,53]		1 [55]	1 [51]					
Hypomanic episodes		1 [60]																
Mixed episodes		1 [51]	1 [66]	1 [47]					1 [67]									
Bipolar depression	3 [29,51,58]				1 [67]			1 [57]		1 [66]								
Unipolar depression	1 [58]		2 [48,49]								2 [29,51]							1 [45]
Schizophrenia	1 [71]	1 [72]	5 [29,48,49,63,70]					1 [68]							1 [69]			1 [45]
Schizoaffective					1 [70]			1 [50]										
Total	N = 7	N = 9	N = 15	N = 2	N = 2	N = 1		N = 6	N = 1	N = 5	N = 2	N = 1	N = 1		N = 1			N = 2
Total sum	N = 16		N = 17		N = 3		N = 6		N = 6		N = 3		N = 1		N = 1		N = 2	

## Data Availability

Data are not shared due to ethical issues.
